# A Dual-Band, Dual-Polarized Filtering Antenna Based on Cross-Shaped Dielectric Strip Resonator

**DOI:** 10.3390/mi13122069

**Published:** 2022-11-25

**Authors:** Lixin Jin, Hui Tang, Jin Shi, Longlong Lin, Kai Xu

**Affiliations:** 1 School of Information Science and Technology, Nantong University, Nantong 226019, China; 2 Research Center for Intelligent Information Technology, Nantong University, Nantong 226019, China; 3 Nantong Key Laboratory of Advanced Microwave Technology, Nantong University, Nantong 226019, China; 4 Zhongtian Radio Frequency Cable Co., Ltd., Nantong 226010, China

**Keywords:** dual-band, dual-polarized, cross-shaped dielectric strip resonator, dual-mode microstrip resonator, filtering antenna

## Abstract

A dual-band, dual-polarized filtering antenna with a cross-shaped dielectric strip resonator is proposed. The dual-band filtering radiation function is achieved by utilizing the odd and even modes of the stub loaded microstrip resonator to excite the TM_δ1_ and TM_δ3_ mode in each polarization direction of the cross-shaped dielectric strip resonator. The cross-shaped dielectric strip resonator is synthesized by the E-field distributions and the magnitude comparison along different polarization directions, which can ensure the isolation between two polarizations. Compared with dual-band filtering dielectric antennas, the proposed antenna has the characteristic of dual-polarized radiation, as well as a low profile. A prototype is fabricated and measured, which operates at 3.5 GHz and 4.9 GHz with the fractional bandwidths (FBW) of 5.40% and 2.03%, respectively, and the gains of these two bands are 6.4 dBi and 6.2 dBi, respectively. The two radiation nulls are located at 4.4 GHz and 5.1 GHz. Furthermore, the measured isolation between the two ports in the frequency band can achieve 16 dB.

## 1. Introduction

With the advances in wireless communication technology, antennas are widely used as a radio frequency front end for transmitting and receiving signals. Among them, the filtering dielectric antenna [1,2,3,4,5,6] has attracted much attention because it has both the advantages of filtering antenna that reduce the filter requirements in the system and effectively reduces the mutual coupling between two antennas working in adjacent frequency bands. It also has the advantages of dielectric antenna with low loss, high efficiency, and high design freedom. The dual-band antenna [7,8] and dual-polarized antenna [9,10,11] reduce the size of the system, reduce the cost, and improve the communication efficiency from the aspects of frequency band number and polarization number, respectively. Therefore, it is valuable to design a dual-band, dual-polarized filtering dielectric antenna.

In accordance with research, the majority of the existing dual-band, dual-polarized filtering antennas are ground on metal radiators [12,13,14,15,16,17,18,19]. In [12], an upper-band filtering cross dipole is embedded into a lower-band filtering magnetoelectric dipole for realizing the dual-band, dual-polarized filtering operation. In [13], through open-circuit, stepped-impedance resonators added microstrip line to feed four slots etched patch, a ±45° polarized dual-band filtering patch antenna is realized. In [14], with the introduction of a vertical corner pin inside the cavity, TE210 mode and TE120 mode radiation frequencies can be controlled to realize dual-band operation. In [15], by combining a cross slot pair, four shorting pins, and four parasitic strips, a band-notched property between two operation bands is obtained. In [16], by using the C-shaped and L-shaped filtering structure to feed lower-band and upper-band dipoles, a dual-band filtering response with dual-polarized radiation is achieved. In [17], by employing a dual-mode, stub-loaded resonator to feed the X-band and C-band patches, the dual-band filtering radiation function and dual-polarized radiation are realized at the same time. In [18], the dual-band, dual-polarized filtering characteristics are performed by placing four upper-band patch antennas on the lower-band slot antenna and using microstrip lines in the radiating slots to couple to the open-ended stubs on the feeder. In [19], dual-band, dual-polarized characteristics are achieved by using two fully-shielded, quarter-mode substrate-integrated waveguide cavities to excite the upper-band and lower-band patches.

The dual-band filtering dielectric antenna reported is only single-polarized [20,21,22,23]. In [20], the dielectric resonator and the slot lines contribute to two radiation frequency bands, and the open stubs of the feedline and the coupled slot lines accomplish three radiation nulls. In [21], the dual-mode dielectric radiator uses the quadruple-mode, stub-loaded loop resonator to feed, and realizes the dual-band filtering characteristics. In [22], the dual-mode dielectric resonator uses a microstrip line feed to realize the dual-band filtering design with higher frequency selectivity. In [23], the dual-band characteristic is achieved by even-mode coupling between three dielectric strip resonators. The odd-mode coupling between the dielectric strip resonators produces two radiation nulls. The above-mentioned dual-band, single-polarized filtering dielectric antennas have directional radiation, and in addition, some omnidirectional radiation dual-band, single-polarized filtering dielectric antennas are reported [24,25,26]. As a conclusion, to our knowledge, no dual-band, dual-polarized filtering dielectric antennas have been reported yet.

In this paper, a dual-band, dual-polarized filtering dielectric antenna utilized by the cross-shaped dielectric strip resonator is proposed for the first time. The TM_δ1_ and TM_δ3_ modes of each polarization direction for the cross-shaped dielectric strip resonator are excited through the odd and even modes of the stub-loaded microstrip resonator, thereby realizing the dual-band filtering operation. The electric field distribution of the TM_δ1_ mode (TM_δ3_ mode) is concentrated in one polarization direction of the cross-shaped dielectric strip resonator, while that of the other polarization direction is weak, thus ensuring the isolation of the two polarizations. The dielectric resonator and the microstrip resonator are analyzed and a prototype is designed and compared with the existing works.

## 2. Proposed Dual-Band, Dual-Polarized Filtering Antenna Based on Cross-Shaped Dielectric Strip Resonator

**Figure 1:**
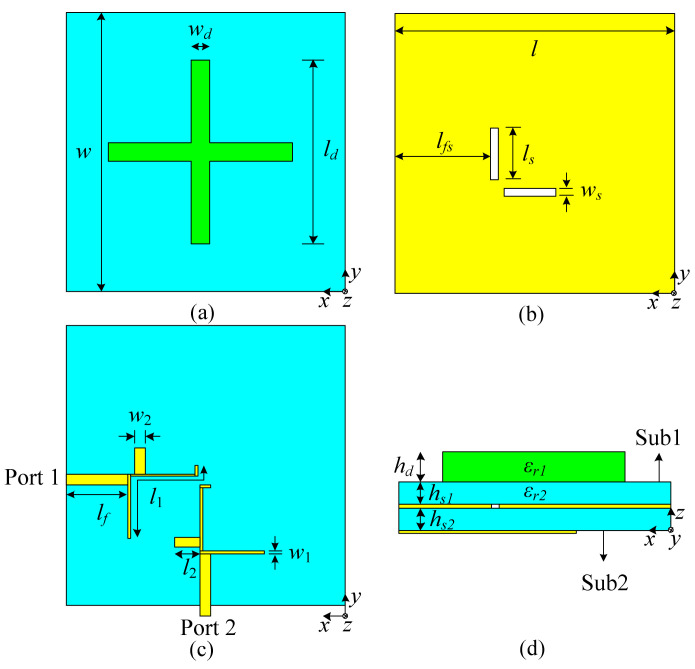
Configuration of the proposed antenna: (**a**) top layer; (**b**) middle layer; (**c**) bottom layer; (**d**) side view.

As shown in Figure 1, the proposed dual-band, dual-polarized filtering dielectric antenna consists of a cross-shaped dielectric strip resonator at the top layer of substrate 1, a horizontal slotted and vertical slotted ground are located in the middle of substrate 1 and substrate 2, and two middle-loaded, open-circuit stub dual-mode microstrip resonators are located on the bottom layer. The cross-shaped dielectric strip resonator is on ceramic sheets (*ε_r_*_1_ = 89.5 and tan *δ*_1_ = 0.0006), while two substrates are RO4003C substrates (*ε_r_*_2_ = 3.38 and tan *δ*_2_ = 0.0027). The full-wave simulation is undertaken using computer simulation technology.

### 2.1. Cross-Shaped Dielectric Strip Resonator

**Figure 2:**
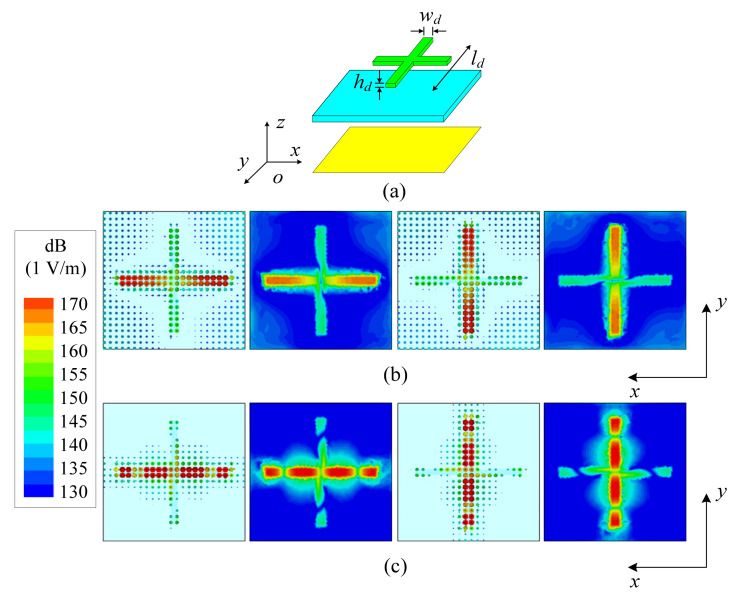
Configuration and E-field distributions of the cross-shaped dielectric strip resonator in *xoy*-plane: (**a**) configuration; (**b**) TM_δ1_ mode; (**c**) TM_δ3_ mode.

Figure 2a shows the structure of the cross-shaped dielectric strip resonator, which is mainly composed of a cross-shaped dielectric strip with a high relative permittivity (*ε_r_*_1_) of 89.5 on the top layer. Located in the middle layer is a substrate with low relative permittivity (*ε_r_*_2_) of 3.38, and the bottom layer is a metal ground. Figure 2b,c shows the electric field distribution of the TM_δ1_ and TM_δ3_ modes of the cross-shaped dielectric strip resonator, respectively. As shown from Figure 2b, the resonant mode is mainly the TM_δ1_ mode on the dielectric strip resonators in horizontal and vertical directions, respectively, and when the electric field distribution is gathered on the dielectric strip resonators in the horizontal (vertical) direction, the electric field allocation of the dielectric strip resonators in the perpendicular vertical (horizontal) direction is weak, thus ensuring the isolation of the TM_δ1_ mode when motivating the two mutual vertical directions. Similarly, it is observed in Figure 2c that the resonant mode is mainly the TM_δ3_ mode on the horizontal and vertical dielectric strip resonators, respectively. When the electric field distribution is gathered on the dielectric strip resonators in the horizontal (vertical) direction, the electric field allocation of the dielectric strip resonators perpendicular to the vertical (horizontal) direction is weak, thus ensuring the isolation of TM_δ3_ mode in the two mutually vertical directions.

**Figure 3:**
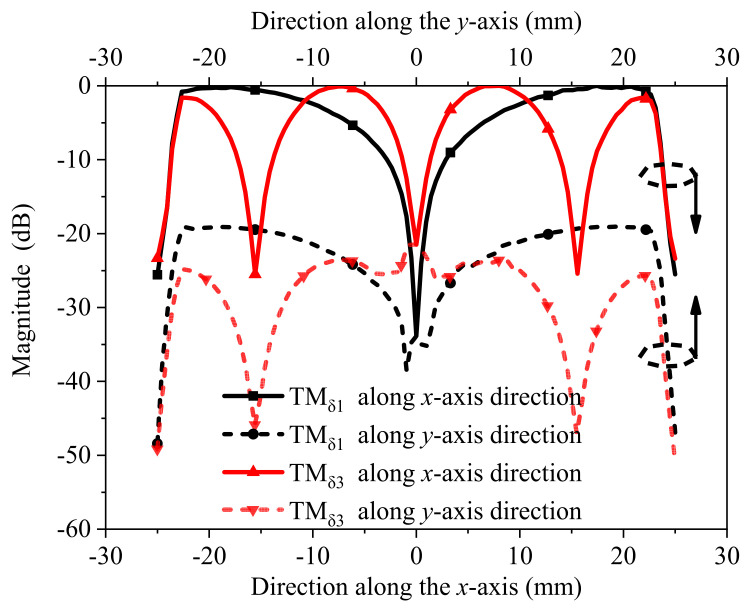
The magnitude of the electric fields distributed by the TM_δ1_ and TM_δ3_ modes along the *x*-axis and along the *y*-axis.

Figure 3 shows that the magnitude of the electric fields distributed by TM_δ1_ mode along the *x*-axis and along the *y*-axis differs by 18 dB and the isolation of TM_δ1_ mode in the two mutually perpendicular directions is ensured. Similarly, TM_δ3_ mode along the *x*-axis and along the *y*-axis differs by 23 dB and the isolation of the TM_δ3_ mode in the two mutually perpendicular directions is ensured. As can be seen from Figure 3, when the horizontal dielectric strip resonator is motivated, the dielectric strip resonator in the vertical direction is not motivated.

As the proposed cross-shaped dielectric strip resonator meets the condition of the equivalent dielectric waveguide model, it can be analyzed as a dielectric waveguide model. When the electrical lengths are *λ_g_*/2 and 3*λ_g_*/2, the cross-shaped dielectric strip resonator resonates at TM_δ1_ mode and TM_δ3_ mode, respectively [27,28]. Therefore, the resonant frequency of TM_δ1_ mode or TM_δ3_ mode for the cross-shaped dielectric strip resonator can be calculated as follows:

(1) $$f_{0}=\frac{\theta /l_{d}+\left(\beta_{y}^{2}+\beta_{z}^{2}\right)}{2\pi \sqrt{\mu \varepsilon_{0}\varepsilon_{reff}}}$$

where *θ* represents the electric size of the cross-shaped dielectric strip resonator, *ε_reff_* = [(*ε_r_*_1_ × *h_d_*) + (*ε_r_*_2_ × *h_s_*_1_)]/(*h_d_* + *h_s_*_1_) is the effective relative permittivity of the proposed cross-shaped dielectric strip resonator, and *β_y_* and *β_z_* can be obtained [27] as:

(2) $$\beta_{y}\left(\frac{w_{d}}{2}\right)=\tan^{-1}\left(\sqrt{\beta_{0}^{2}\left(\varepsilon_{reff}-\varepsilon_{r2}\right)-\beta_{y}^{2}}/\beta_{y}\right)$$

(3) $$\beta_{z}=\pi /2h_{eff}$$

where *β*_0_ is the propagation constant in the vacuum, and *h_eff_* = *h_s_*_1_ + *h_d_* is the effective height of the proposed cross-shaped dielectric strip resonator. By combining Equations (1) and (3), the size parameter of the cross-shaped dielectric strip resonator can be obtained.

### 2.2. Dual-Mode Microstrip Resonator

**Figure 4:**
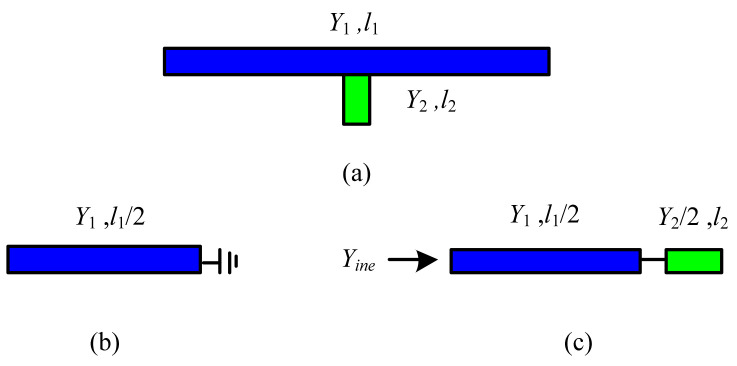
Configuration of the dual-mode microstrip resonator: (**a**) configuration; (**b**) odd-mode circuit model; (**c**) even-mode circuit model.

Figure 4a shows a dual-mode microstrip resonator with open stubs loaded in the middle. On account of the symmetry of the structure, it can be analyzed through the even-odd-mode equivalent circuit [29,30]. When the resonator resonates in an odd mode, it is equivalent to an ideal electric wall along the symmetry plane of the resonator, and the odd-mode equivalent circuit shown in Figure 4b is obtained. The resonance conditions at this time are:

(4) $$f_{odd}=\frac{c}{2l_{1}\sqrt{\varepsilon_{r2}}}$$

where *c* is the transmission velocity of the free-space wave, *ε_r_*_2_ is the relative permittivity of substrate 2, and *f_odd_* is the odd-mode resonance frequency of the dual-mode microstrip resonator.

Similarly, when the resonator resonates in the even mode, it is equivalent to an ideal magnetic wall along the symmetry plane of the resonator. The even mode equivalent circuit is shown in Figure 4c. The resonance conditions at this time are:

(5) $$\frac{Y_{2}}{2}\tan(\frac{2\pi f_{even}l_{2}\sqrt{\varepsilon_{r2}}}{c})+Y_{1}\tan(\frac{\pi f_{even}l_{1}\sqrt{\varepsilon_{r2}}}{c})=0$$

where *f_even_* is the even-mode resonance frequency of the dual-mode microstrip resonator. By combining Equations (4) and (5), the size parameter of the dual-mode microstrip resonator can be obtained for the resonant frequency requirements of odd and even mode.

### 2.3. Parametric Study of l_s_, w_s_, and l_fs_

**Figure 5:**
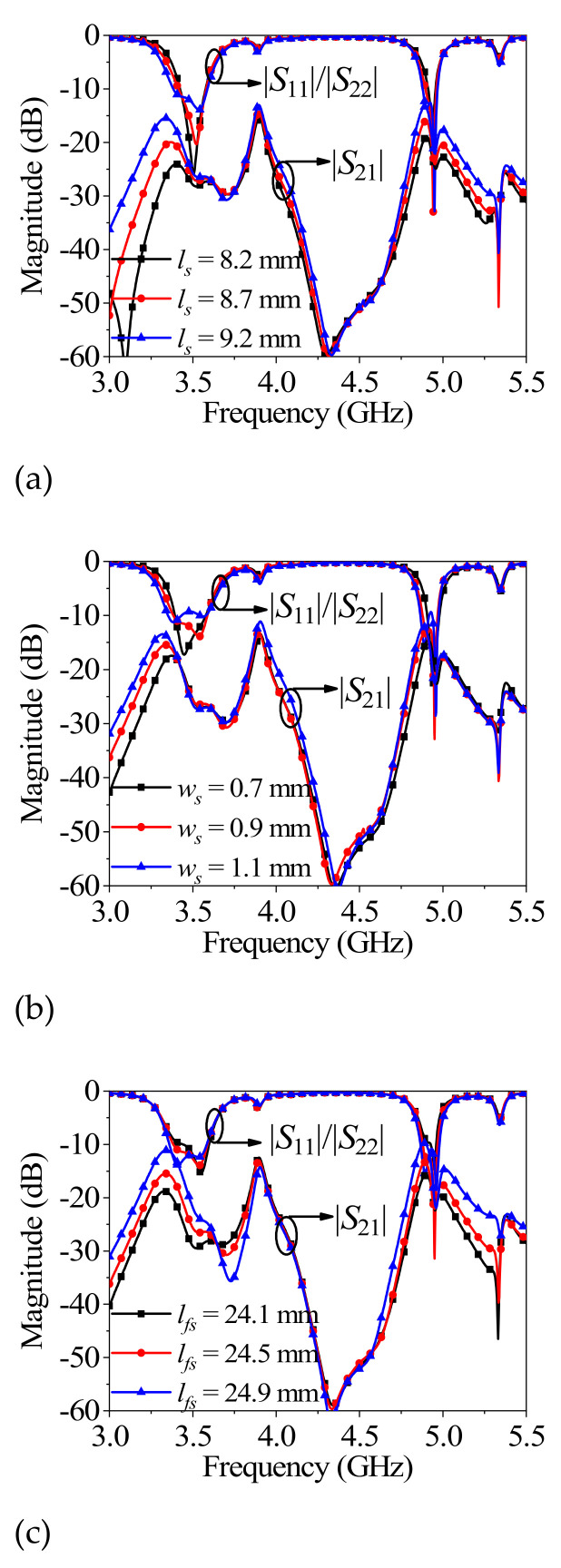
The simulated performance variation with different parameters: (**a**) different *l_s_*; (**b**) different *w_s_*; (**c**) different *l_fs_*.

Figure 5 depicts the effects of *l_s_*, *w_s_*, and *l_fs_* on antenna performance. It can be seen from Figure 5a,b that the bandwidth of two radiation bands increases, and the isolation between the two ports decreases with the increase in *l_s_* and *w_s_*. When the coupling strength between the cross-shaped dielectric strip resonator and the dual-mode microstrip resonator increases as *l_s_* and *w_s_* increase, the bandwidth of two radiation bands increases. However, the isolation between the different ports decreases because the spacing between paths via two ports decreases as *l_s_* and *w_s_* increase. Figure 5c exhibits that the isolation between the different ports decreases and the impedance matching improves with the increase in *l_fs_*. The isolation decreases because the spacing between paths via two ports decrease as *l_fs_* increases. The impedance matching improves because the feeding position becomes more suitable with the increase in *l_fs_*.

### 2.4. Design Procedure 

The design guidelines of the dual-band, dual-polarized filtering antenna based on cross-shaped dielectric strip resonator are described below.

(1) On the basis of Equations (1) and (3), the initial dimensions of *l_d_*, *w_d_,* and *h_d_* can be calculated. The initial dimensions of *l*_1_, *l*_2_, *Y*_1_, and *Y*_2_ in the dual-mode microstrip resonators are obtained based on Equations (4) and (5), and then the initial values of *w*_1_ and *w*_2_ are obtained according to *Y*_1_ and *Y*_2_.

(2) According to the variation law in Figure 5a,b, the appropriate initial size of *l_s_* and *w_s_* can be obtained based on the required bandwidth and isolation.

(3) Finally, according to the variation law in Figure 5c, tuning the parameter *l_fs_* provides a good matching performance.

## 3. Results

**Figure 6:**
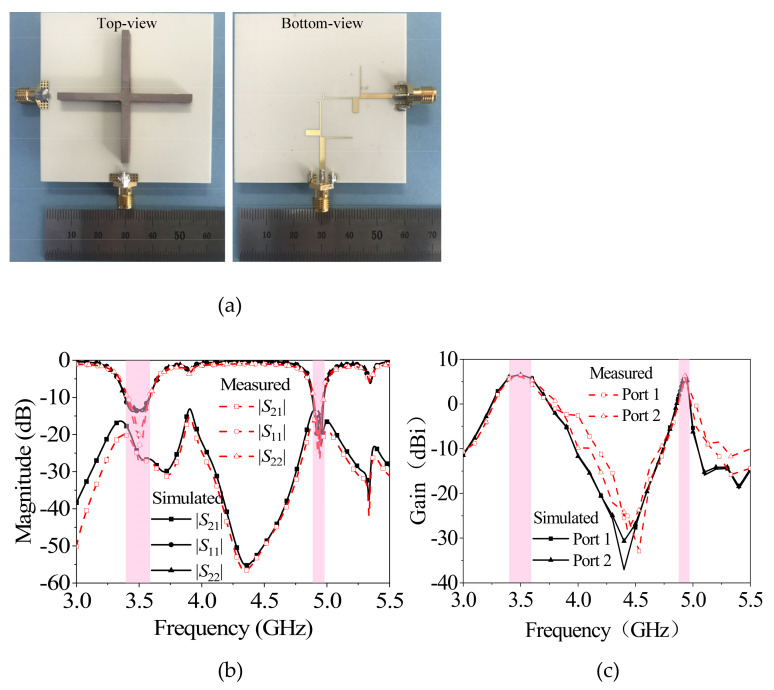
Photographed, simulated, and measured results of the proposed antenna: (**a**) photograph; (**b**) |S_21_|, |S_11_| and |S_22_|; (**c**) gain.

Based on the above design process of the dual-band, dual-polarized filtering antenna based on a cross-shaped dielectric strip resonator, a prototype as shown in Figure 6a was fabricated and tested. Based on the above analysis, the specific sizes of the antenna were: *l* = 60 mm, *w* = 60 mm, *l_d_* = 45.5 mm, *w_d_* = 2.5 mm, *h_d_* = 3.9 mm, *l_s_* = 9.2 mm, *w_s_* = 0.9 mm, *l*_1_ = 26.2 mm, *w*_1_ = 0.3 mm, *l*_2_ = 5 mm, *w*_2_ = 2 mm, *l_f_* = 15.8 mm, *l_fs_* = 24.5 mm, *h_s_*_1_ = 0.913 mm, *h_s_*_2_ = 0.813 mm, *h*_1_ = 0.913 mm, and *h*_2_ = 0.813 mm.

Figure 6b,c shows the simulated and measured |S_11_| and gain of the prototype. The 10-dB impedance-matching bandwidths of two operating bands are 5.40% (at 3.5 GHz) and 2.03% (at 4.9 GHz), respectively. The measured gains in two bands are 6.4 dBi and 6.2 dBi, respectively. Two radiation nulls occur at 4.4 GHz and 5.1 GHz, respectively. The measured isolation between the two ports in the frequency band is greater than 20 dB and 16 dB, respectively. At the same time, it can be seen that the S-parameter curves and gain curves of the two ports are basically consistent, indicating that the two polarizations have good consistency.

**Figure 7:**
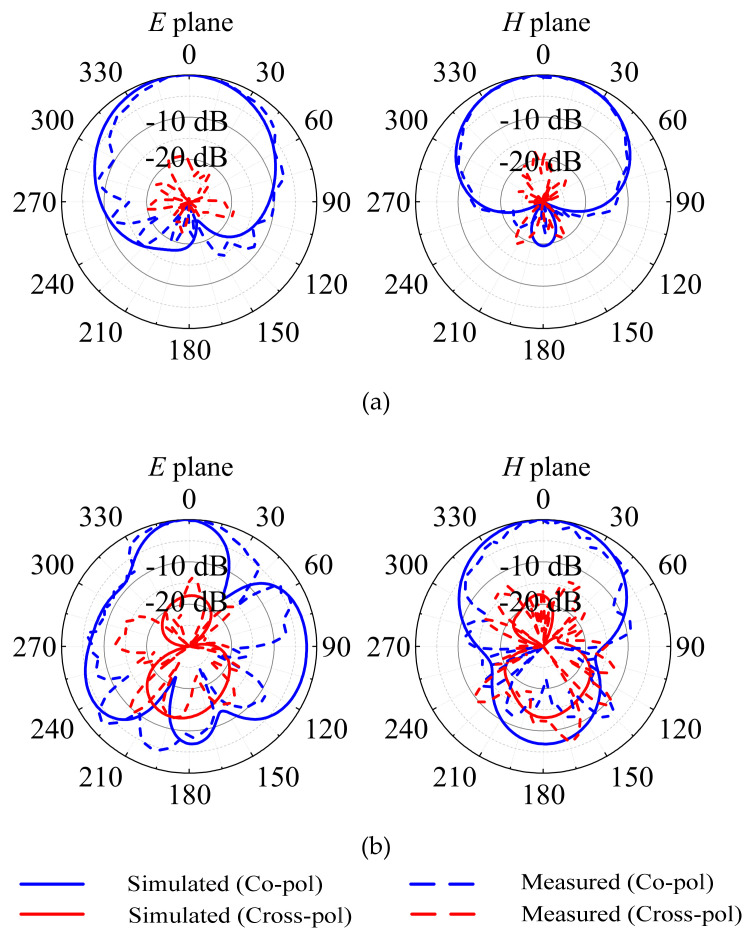
Simulated and measured radiation patterns of the proposed design at excitation port 1: (**a**) at 3.5 GHz; (**b**) at 4.9 GHz.

The patterns of the E and H planes at 3.5 GHz and 4.9 GHz, respectively, when port 1 is excited are shown in Figure 7a,b. Figure 7a shows that when the antenna is operating at 3.5 GHz, the measured 3-dB beamwidths of the E- and H-plane patterns are approximately 77° and 78°, respectively. The cross-polarization level is also less than −19 dB within the 3-dB beamwidth of the E plane and less than −18 dB within the 3-dB beamwidth of the H plane. In the E and H planes, the front-to-back ratio is recorded at 21.7 dB and 22.7 dB, respectively. Figure 7b shows that when the antenna is operating at 4.9 GHz, the measured 3-dB beamwidths of the E- and H-plane patterns are approximately 53° and 49°, respectively. The cross-polarization level is also less than −14 dB within the 3-dB beamwidth of the E plane and less than −14 dB within the 3-dB beamwidth of the H plane. In the E and H plane, the front-to-back ratio is recorded at 10 dB and 19 dB, respectively.

**Figure 8:**
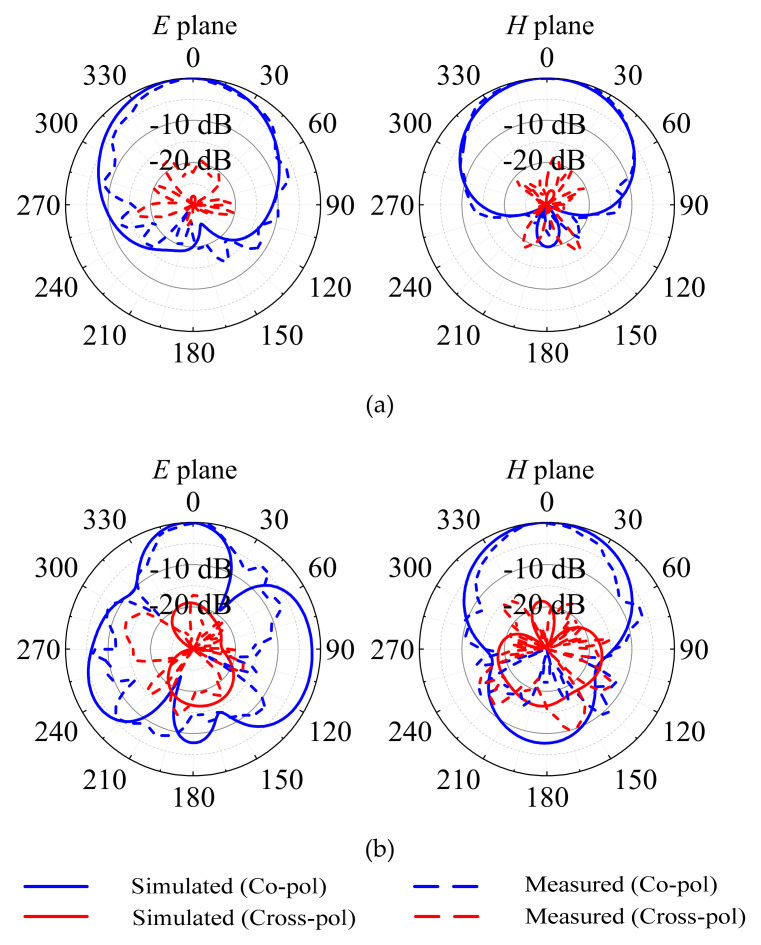
Simulated and measured radiation patterns of the proposed design at excitation port 2: (**a**) at 3.5 GHz; (**b**) at 4.9 GHz.

Similarly, the patterns of the E and H planes at 3.5 GHz and 4.9 GHz, respectively, when port 2 is excited are shown in Figure 8a,b. Figure 8a shows that when the antenna is operating at 3.5 GHz, the measured 3-dB beamwidths of the E- and H-plane patterns are approximately 69° and 85°, respectively. The cross-polarization level is also less than −19.7 dB within the 3-dB beamwidth of the E plane and less than −18.3 dB within the 3-dB beamwidth of the H plane. In the E and H planes, the front-to-back ratio is recorded at 21.3 dB and 22.7 dB, respectively. Figure 8b shows that when the antenna is operating at 4.9 GHz, the measured 3-dB beamwidths of the E- and H-plane patterns are approximately 34° and 57°, respectively. The cross-polarization level is also less than −17.0 dB within the 3-dB beamwidth of the E plane and less than −18.4 dB within the 3-dB beamwidth of the H plane. In the E and H plane, the front-to-back ratio is recorded at 15.3 dB and 21.2 dB, respectively.

The performances of the proposed work and the state-of-the-art works are summarized in Table 1. Contrasted with the dual-band, dual-polarized metal filtering antenna [13,16,17,18,19], the proposed antenna has the features of low conductor loss and higher design freedom. Contrasted with the dual-band filtering dielectric antennas [20,21,22,23], the proposed antenna not only has the function of dual-polarized radiation, but also has the advantage of a low profile.

## 4. Conclusions

In this paper, a dual-band, dual-polarized filtering antenna based on a cross-shaped dielectric strip resonator is proposed. The dual-band filtering radiation function is realized by utilizing the odd and even modes of the stub-loaded microstrip resonator to excite the TM_δ1_ and TM_δ3_ modes in each polarization direction of the cross-shaped dielectric strip resonator. A prototype is fabricated and measured, which operates at 3.5 GHz and 4.9 GHz with the fractional bandwidths (FBW) of 5.40% and 2.03%, respectively, and the gains of these two bands are 6.4 dBi and 6.2 dBi, respectively. The two radiation nulls are located at 4.4 GHz and 5.1 GHz. Furthermore, the measured isolation between the two ports in the frequency band can achieve 16 dB. Contrasted with the state-of-art designs, the proposed antenna has all the considerations of dual-polarized radiation, low conductor loss, and a low profile.

## Figures and Tables

**Table 1:** The proposed antenna compared with other related works.

Ref. No	*f_0_*(GHz)	Radiator Size	10-dB FBW(%)	Gain	Radiator Type	Polarization
		(λ_0_ × λ_0_ × λ_0_)		(dBi)		
[13]	3.50/4.90	0.31 × 0.29 × 0.13	12.30/7.60	8.34/8.70	Metal	Dual
[16]	2.60/3.45	1.22 × 1.22 × 0.11	7.60/8.60	8.50/8.10	Metal	Dual
[17]	5.20/9.90	0.25 × 0.25 × 0.06	4.60/7.00	10.10/12.50 (2 × 2)	Metal	Dual
[18]	0.83/3.55	0.29 × 0.29 × 0.14	32.50/8.40	8.30/8.30	Metal	Dual
[19]	25.83/38.46	0.39 × 0.39 × 0.14	13.00/9.20	5.40/5.60	Metal	Dual
[20]	3.50/5.06	0.24 × 0.24 × 0.18	7.75/1.50	5.50/6.75	Dielectric	Single
[21]	3.42/5.28	0.46 × 0.34 × 0.22	5.80/4.00	5.18/6.45	Dielectric	Single
[22]	1.76/2.02	0.47 × 0.17 × 0.10	5.30/6.40	5.58/6.62	Dielectric	Single
[23]	7.70/13.35	0.77 × 0.77 × 0.06	11.40/4.80	7.65/10.50	Dielectric	Single
This work	3.50/4.90	0.53 × 0.53 × 0.06	5.40/2.03	6.40/6.20	Dielectric	Dual

## Data Availability

The data presented in this study are available on request from the corresponding author.
